# Hemodynamics are associated with subsequent lumen remodeling and clinical maturation of hemodialysis arteriovenous fistula

**DOI:** 10.1038/s41598-025-89896-z

**Published:** 2025-02-19

**Authors:** Yong He, Guo Wei, Tom Greene, Peter B. Imrey, Hannah Northrup, Milena K. Radeva, Gerald J. Beck, Jennifer J. Gassman, Larry W. Kraiss, Michelle Robbin, Prabir Roy-Chaudhury, Alfred K. Cheung, Scott A. Berceli, Yan-Ting Shiu, the Hemodialysis Fistula Maturation (HFM) Study Group, T. Huber, S. Berceli, M. Jansen, G. McCaslin, Y. Trahan, A. Cheung, L. Kraiss, T. Greene, D. Kinikini, G. Treiman, D. Ihnat, M. Sarfati, I. Lavasani, M. Maloney, L. Schlotfeldt, G. Beck, J. Gassman, P. Imrey, L. Li, J. Alster, M. Li, J. MacKrell, M. Radeva, B. Weiss, K. Wiggins, H. Feldman, L. Dember, A. Farber, J. Kaufman, L. Stern, P. LeSage, C. Kivork, D. Soares, M. Malikova, J. Vita, N. Hamburg, M. Duess, A. Levit, M. Allon, C. Young, M. Taylor, L. Woodard, K. Mangadi, P. Roy-Chaudhury, R. Munda, T. Lee, R. Alloway, M. El-Khatib, T. Canaan, A. Pflum, L. Thieken, B. Campos-Naciff, M. Vazquez, W. Vongpatanasin, I. Davidson, C. Hwang, T. Lightfoot, C. Livingston, A. Valencia, B. Dolmatch, A. Fenves, N. Hawkins, J. Himmelfarb, C. Alpers, K. Hudkins, T. Wietecha, C. Buchanan, C. Clark, C. Crawford, J. Hamlett, J. Kundzins, L. Manahan, J. Wise, H. Higgins, S. Ke, O. Mandaci, C. Snell, J. Gravley, S. Behnken, R. Mortensen, J. Kusek, R. Star, G. Chertow, A. Besarab, K. Brayman, M. Diener-West, T. Louis, D. Harrison, L. Inker, W. McClellan, J. Rubin

**Affiliations:** 1 Division of Vascular Surgery and Endovascular Therapy, University of Florida, Gainesville, FL USA; 2 Division of Nephrology and Hypertension, University of Utah, 30 N Mario Capecchi Driver, 3Rd Floor South, Salt Lake City, UT 84112 USA; 3 Division of Epidemiology, University of Utah, Salt Lake City, UT USA; 4 Department of Population Health Sciences, University of Utah, Salt Lake City, UT USA; 5 Department of Quantitative Health Sciences, Cleveland Clinic, Cleveland, OH USA; 6 Department of Medicine, Cleveland Clinic Lerner College of Medicine of Case Western Reserve University, Cleveland, OH USA; 7 Mellon Center for Multiple Sclerosis Treatment and Research, Cleveland Clinic, Cleveland, OH USA; 8 Division of Vascular Surgery, University of Utah, Salt Lake City, UT USA; 9 Department of Radiology, University of Alabama at Birmingham, Birmingham, AL USA; 10 Division of Nephrology and Hypertension, University of North Carolina, Chapel Hill, NC USA; 11 Department of Medicine, W. G. (Bill) Hefner Veterans Affairs Medical Center, Salisbury, NC USA; 12 Vascular Surgery Section, Malcom Randall Veterans Affairs Medical Center, Gainesville, FL USA; 13 Renal Section, Veterans Affairs Salt Lake City Healthcare System, Salt Lake City, UT USA; 14 Nora Eccles Harrison Cardiovascular Research and Training Institute, University of Utah, Salt Lake City, UT USA; 15 University of Pennsylvania, Philadelphia, PA USA; 16 Boston University, Boston, MA USA; 17 University of Alabama, Birmingham, AL USA; 18 University of Cincinnati, Cincinnati, OH USA; 19 University of Texas Southwestern, Dallas, TX USA; 20 University of Washington, Seattle, WA USA; 21 National Institute of Diabetes and Digestive and Kidney Diseases (NIDDK), Bethesda, MD USA; 22 Stanford University, Stanford, CA USA; 23 University of Virginia, Charlottesville, VA USA; 24 Johns Hopkins University, Baltimore, MD USA; 25 Temple University, Philadelphia, PA USA; 26 Tufts University, Medford, MA USA; 27 Emory University, Atlanta, GA USA; 28 Georgetown University, Washington, DC USA

**Keywords:** Medical research, Nephrology

## Abstract

The pathogenesis of arteriovenous fistula (AVF) maturation failure is unclear. We evaluated the associations of wall shear stress (WSS) with subsequent AVF remodeling and clinical maturation using regression models in this prospective cohort study. Participants underwent duplex ultrasound at postoperative Day 1, Week 2, and Week 6 to measure AVF blood flow rate and diameter of the draining vein and proximal artery. The median vein WSS of 602 participants decreased from Day 1 to Week 6 (from 33.4 to 21.6 dyne/cm^2^) but did not change noticeably for the artery (from 58.4 to 55.1 dyne/cm^2^). WSS was positively associated with subsequent lumen expansion, with doubling of Day-1 WSS presaging a 9% (95% confidence interval (CI) 5%-14%; P < 0.001) greater Day 1-to-Week 6 increase in vein lumen cross-sectional area and a 5% (95% CI: 1%-10%; P = 0.020) greater increase in artery lumen area. The odds of unassisted clinical maturation increased by 45% (95% CI: 11%-89%; P = 0.006) with each doubling of Day-1 vein WSS, and by 82% (95% CI: 39%-250%; P < 0.001) with each doubling of Day-1 artery WSS. These findings show that WSS was positively associated with subsequent lumen expansion and AVF unassisted clinical maturation.

## Introduction

A mature arteriovenous fistula (AVF) is the preferred vascular access for maintenance hemodialysis in patients with end-stage kidney disease due to a lower complication rate than synthetic arteriovenous grafts or central venous catheters^1,2^ Maturation requires sufficient vascular wall remodeling, which leads to increases in lumen cross-sectional area of the AVF vein and blood flow rate. Immediately after AVF creation, the flow rate increases many folds due to bypassing of distal high-resistance vasculature by direct anastomosis between a native artery and vein^3^ The vein diameter is also immediately distended by an artery-level blood pressure. Further, sufficient increases in lumen diameter and blood flow rate are needed for clinical use of the AVF. However, up to 60% AVFs do not adequately mature without additional interventions^4–6^.

Understanding the mechanisms that determine AVF maturation is important for developing strategies to improve it. The 7-center, prospective Hemodialysis Fistula Maturation (HFM) study investigated the associations of clinical, anatomical, biological, and process-of-care attributes with AVF development and maturation^7^. Many important findings from the HFM Study have been reported^4,8–16^. Hemodynamic factors have been demonstrated to regulate the structure and function of arteries and vein bypass grafts^17–21^. Therefore, hemodynamic factors are also likely to regulate AVF remodeling. Wall shear stress (WSS), induced by viscous flowing blood on the luminal surface of a blood vessel, is the mostly studied hemodynamic factor and increases remarkably in an AVF compared to native vessels. Indeed, in an HFM ancillary study, we found that a higher WSS was associated with a larger lumen expansion at later time points even under a chronic uremic condition^22^. In that ancillary study, magnetic resonance imaging (MRI) was performed at postoperative Day 1, Week 6, and Month 6 to acquire the three-dimensional lumen geometry and blood flow rates, which were then used to derive detailed WSS data via computational fluid dynamics simulation of pulsatile blood flow. However, MRI is not routinely used for the care of dialysis patients and is expensive, and imaging processing and computational fluid dynamics simulation are time consuming, limiting the sample size of our previous study and potential clinical applicability. Ultrasound imaging, the standard of care, was used instead to assess longitudinal AVF lumen diameters and blood flow rates in the parent HFM study.

Here, we use these ultrasound data from the parent HFM study to evaluate the association of WSS with subsequent AVF remodeling, with more patients (602 versus 120) and centers (7 versus 3) and a more clinically relevant imaging modality than our previous study using MRI^8,22^. Importantly, revealing the association of WSS with AVF clinical maturation may directly help manage dialysis patients. Therefore, we also use the HFM study’s rigorously defined and adjudicated clinical maturation data, which were only available for the HFM patients – a distinct minority – in our previous ancillary MRI-based study, to examine the association of WSS with subsequent AVF clinical maturation.

## Methods

### Participants

The overall design and rationale of the HFM study have been published^7^. To be eligible for the study, the patient needed to be on maintenance hemodialysis or expected to initiate hemodialysis within 3 months; scheduled for a single-stage, upper-extremity AVF creation surgery as clinically indicated; not older than 80 years if not yet undergoing chronic dialysis; have a life expectancy of at least 9 months; and willing and able to comply with the required study procedures. This study was approved by the Institutional Review Board at each participating University and performed in accordance with the Declaration of Helsinki. Written informed consent was obtained from all patients.

### Ultrasound and vascular function testing

A preoperative vascular ultrasound scan was performed as routine clinical mapping for AVF creation. The inherent capacity of the artery to dilate likely influences the increases in diameter and flow rate following AVF creation. Therefore, flow-mediated dilation and nitroglycerin-mediated dilation are commonly used in the research setting to assess the capacity of the brachial artery to dilate. These two vascular function tests were performed within 45 days prior to AVF creation surgery on the arm to be used for the AVF creation, unless a patent AVF was already present in that arm. Details of these vascular function tests have been described previously^10^.

Participants also underwent duplex ultrasound at postoperative Day 1, Week 2, and Week 6 to measure AVF blood flow rate at two different positions, 10-cm from the anastomosis in the draining vein and 2-cm from the anastomosis in the proximal artery. Additionally, lumen diameters of the draining vein were measured at positions 2, 5, and 15 cm from the anastomosis, and lumen diameter of the proximal artery was measured 2-cm from the anastomosis. Ultrasound data were extracted from the images by a core facility at the University of Alabama at Birmingham, where sonographers from all clinical centers also received standardized training on performing ultrasound imaging^8^.

### Wall shear stress estimation

Instantaneous WSS varies during a cardiac cycle. Mean WSS in a cycle has been the parameter most commonly used to investigate the role of WSS in vascular biology and disease due to its simplicity and demonstrated importance in regulating vascular structure and function^23–25^. Furthermore, the differences between peak systolic, end-diastolic, and mean WSS are reduced in an AVF due to its low-resistance circuit compared to the normal arterial circulation. Therefore, mean WSS was used in this study. Based on the mean blood flow rate and lumen diameter obtained from ultrasound, we used the Hagen-Poiseuille equation to estimate the mean WSS as

$${\tau }_{w}=32 \bullet \mu \cdot \frac{Q}{\pi \cdot {d}^{3}}$$

where ${\tau }_{w}$ is WSS (dyne/cm^2^), $\mu$ is blood viscosity (0.0035 Pa·s), Q is mean blood flow rate (mL/s), and d is lumen diameter (cm)^26^.

### Clinical maturation

For patients already undergoing chronic dialysis before AVF creation, clinical AVF maturation was defined as use of the AVF with 2 needles for ≥ 75% of hemodialysis sessions during a continuous 4-week period commencing within 9 months of AVF creation surgery, including 4 consecutive sessions with mean blood pump flow > 300 mL/min or, failing that, any session with Kt/V_urea_ ≥ 1.4 or urea reduction ratio ≥ 70%. For patients initiating chronic dialysis more than 9 months after AVF creation, the first dialysis session meeting the blood pump flow, Kt/V_urea_, or urea reduction ratio criteria must have been achieved within 4 weeks of hemodialysis initiation. Clinical maturation was subclassified as assisted if preceded by a percutaneous or surgical intervention to promote AVF maturation, and unassisted otherwise^11^.

### Statistical analysis

#### Missing data

To mitigate bias caused by missing data, we multiply imputed missing vascular function tests, ultrasound measurements for visits scheduled prior to AVF thrombosis or patient death, and demographics. The multiple imputation procedure has been described previously^10^. Ten imputed data sets were generated using the mice R package following a fully sequential imputation approach in which, for each missing value, 10 replacement values were drawn randomly using predictive mean matching for continuous variables and logistic regression for dichotomous variables. The imputed data were then used in statistical modeling by fitting each model separately to each of the 10 imputed data sets and combining the results using Rubin’s formulas^27^.

#### Descriptive analysis

The observed data without imputation were used in descriptive analyses. Baseline patient characteristics, including age, sex, race, diabetes, and dialysis history, were summarized by mean and standard deviation for continuous variables and by number of patients with corresponding proportion of the cohort for categorical variables (Table 1). Distributions of AVF flow rates and vessel diameters were visually portrayed using boxplots.

**Table 1:** Baseline characteristics of the study cohort (n = 602).

Characteristic	Mean (standard deviation) or n (%)
Age at surgery, years	55.2 (13.4)
Female, n (%)	179 (29.7)
Black race, n (%)*	267 (44.4)
Diabetes, n (%)	353 (58.7)
Body mass index, kg/m^2^	30.4 (7.6)
On dialysis at surgery, n (%)	387 (64.3)
History of coronary artery disease, n (%)	156 (25.9)
History of peripheral artery disease, n (%)	91 (15.1)
History of cerebrovascular disease, n (%)	88 (14.6)

*: Data on 4 patients were missing and imputed.

#### Associations of hemodynamics with subsequent AVF remodeling and unassisted clinical maturation

We first explored the factors that contribute to variability in WSS by examining the associations of WSS with demographic factors and preoperative ultrasound measurements, using multiple linear regressions of natural-logarithmically transformed WSS on baseline factors. WSS was natural-logarithmically transformed due to its highly skewed distribution.

We next characterized the associations of WSS with subsequent lumen area changes. Linear models were used to fit the natural-logarithmically transformed ratios between the cross-sectional areas at two given time points (i.e., later/earlier for “Day 1 to Week 2”, “Week 2 to Week 6”, and “Day 1 to Week 6” separately) to WSS at the earlier time point. Four versions of each model were fit with, respectively, no covariate adjustment (Model I), covariate adjustment for baseline age, sex, race, diabetes, dialysis history, clinical center, preoperative artery diameter, preoperative vein diameter, nitroglycerin-mediated dilation, flow-mediated dilation (Table 2), and AVF location (forearm versus upper arm) (Model II), the same vessel’s natural-logarithmically transformed diameter at the period baseline (Model III), and all of these covariates (Model IV).

**Table 2:** Preoperative vascular lumen diameters and vascular function tests.

Preoperative minimal vein diameter, mm	3.0 (1.1)^&^
Preoperative average* vein diameter, mm	3.7 (1.2)
Preoperative artery diameter, mm	3.8 (1.2)
Brachial artery flow-mediated dilation (%)	4.8 (5.0)
Brachial artery nitroglycerin-mediated dilation (%)	7.2 (6.3)

^&^Data are presented as mean (standard deviation).

*The vein diameters were measured at multiple locations of upper arm or forearm and results averaged.

For the vein, the main analyses were performed using the mean of the areas calculated from diameters at the four locations (2, 5, 10, and 15 cm from the anastomosis). To test whether the coefficients relating WSS to the changes in vein areas differed between the four locations, we used mixed effect models with unstructured covariance matrices to jointly relate WSS to the changes in vein area at each of the four locations, while accounting for the correlations between the four measurements of the same participant’s vein. P-values for comparisons were obtained from Wald tests.

We also applied logistic regression to relate WSS to unassisted clinical maturation at each of the three time points (Day 1, Week 2, and Week 6) separately, with adjustment for the aforementioned covariates.

Finally, since WSS was calculated as an explicit function of blood flow rate, which is known to predict AVF maturation^4^, we replicated these analyses with WSS replaced by blood flow rate to demonstrate the similarities and differences of results, and hence the implications of focusing analyses on each of these related measures.

All analyses were performed using STATA version MP 15.1 (StataCorp LLC., College Station, TX). Two-sided α = 0.05 was used for hypothesis testing.

## Results

### Postoperative blood flow rate, diameters, and wall shear stress

**Fig. 1:**
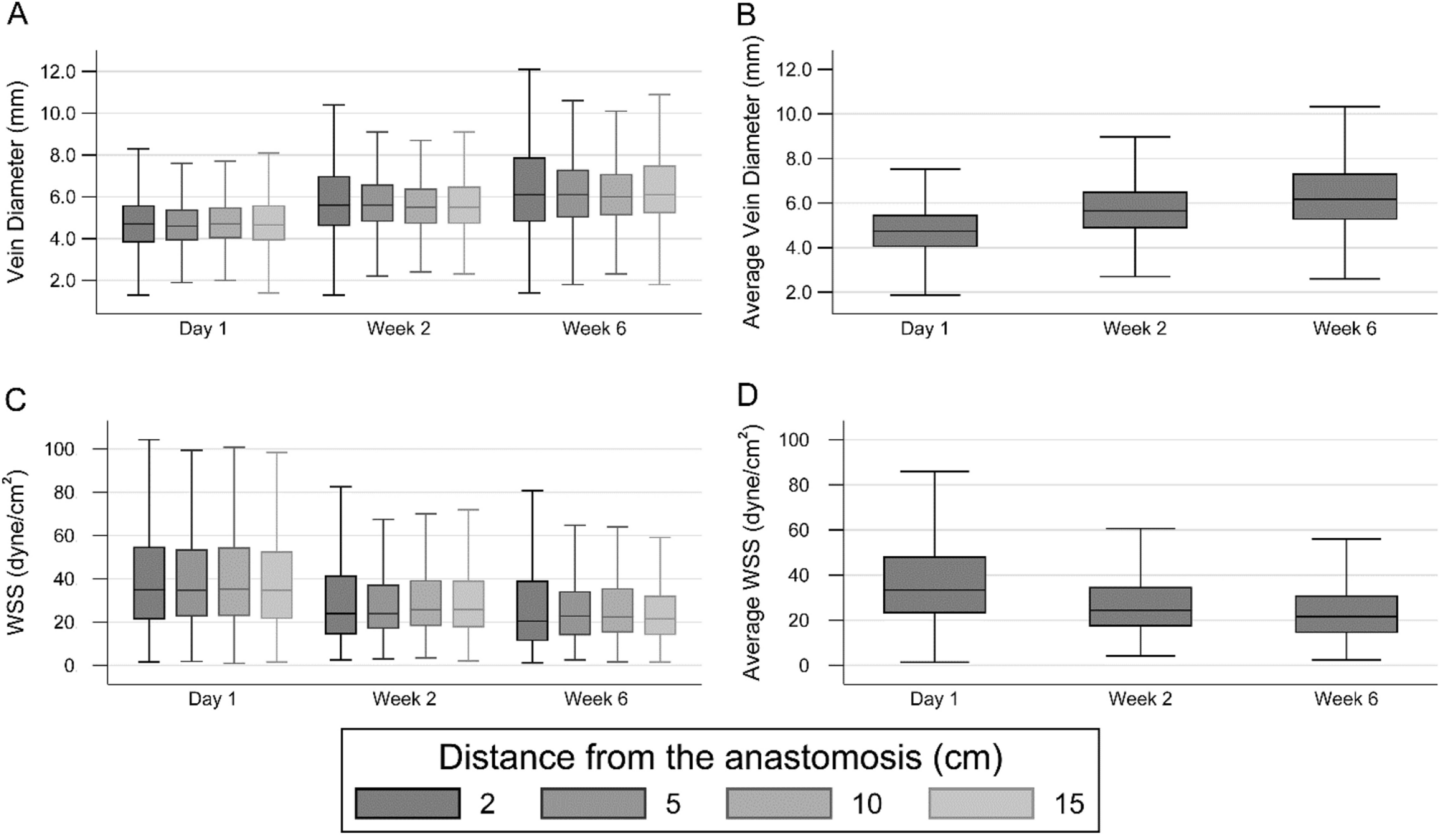
Postoperative fistula vein lumen diameter and wall shear stress (WSS). Box plots of vein diameters at 2, 5, 10, and 15 cm from the anastomosis (panel **A**), diameters averaged over these four locations (panel B), WSS at 2, 5, 10, 15 cm from the anastomosis (panel **C**), and WSS averaged over these four locations (panel D). The whiskers were placed 1.5 times the interquartile range beyond the first and third quartiles, and more extreme values were treated as outliers and not shown.

**Fig. 2:**
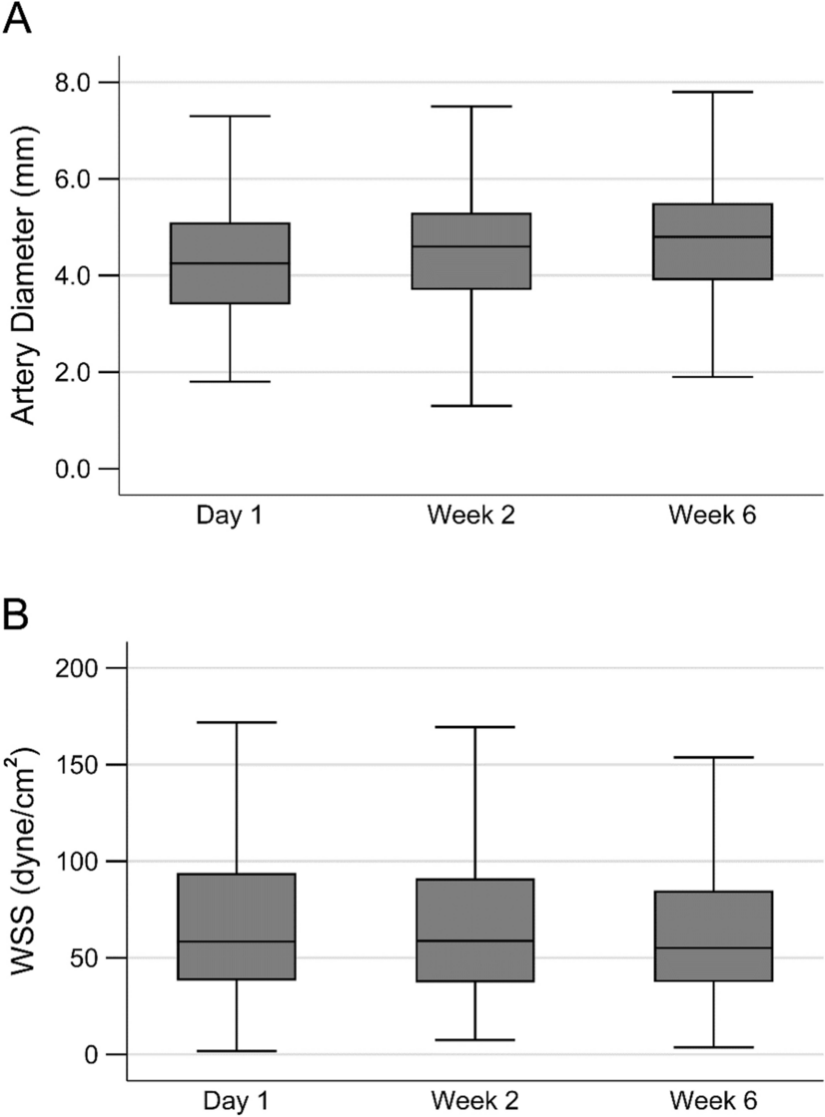
Postoperative fistula artery lumen diameter and wall shear stress (WSS). Box plots of diameters at 2 cm from the anastomosis (panel **A**) and WSS (panel **B**). The whiskers were placed 1.5 times the interquartile range beyond the first and third quartiles, and more extreme values were treated as outliers and not shown.

The imputed preoperative vascular mapping and function test (supplemental Table S1) and postoperative blood flow rate data (Table S2) are similar to the corresponding raw data. The postoperative blood flow rates of the fistula vein and proximal artery increased from Day 1 to Week 6 (Table 3). The fistula vein lumen diameter at each of the 4 measurement sites (Fig. 1A) and their average (Fig. 1B) increased from Day 1 to Week 6, while the fistula vein WSS at each measurement site (Fig. 1C) and their average (Fig. 1D) progressively decreased from Day 1 to Week 6 (with medians 33.4, 24.4, and 21.6 dyne/cm^2^ at Day 1, Week 2, and Week 6, respectively). The fistula vein diameters and WSS values were similar among the four measurement sites, respectively. The proximal artery lumen diameter increased slightly from Day 1 to Week 6 (Fig. 2A), but the WSS values did not change notably (with medians 58.4, 58.8, and 55.1 dyne/cm^2^ at Day 1, Week 2, and Week 6, respectively; Fig. 2B). Table 3 and these box plots demonstrate large variability in flow rate, diameters, and WSS among participants. Even though the artery and vein at the same time point had similar flow rates, the median arterial WSS values exceeded the venous WSS values at the same time point because of the smaller diameter of the artery than the vein. The associations of postoperative vein and artery WSS values with demographics and preoperative ultrasound measurements were evaluated using multiple linear regressions of natural-log transformed WSS and are presented in Tables S3 and S4, respectively.

**Table 3:** Postoperative fistula flow rate data.

Time point	Vessel	Flow rate (mL/min)
Day 1	Vein	692 (434)
	Artery	794 (433)
Week 2	Vein	894 (519)
	Artery	981 (491)
Week 6	Vein	1026 (621)
	Artery	1068 (552)

Data are presented as mean (standard deviation).

### Associations of hemodynamics with subsequent lumen area change

**Fig. 3:**
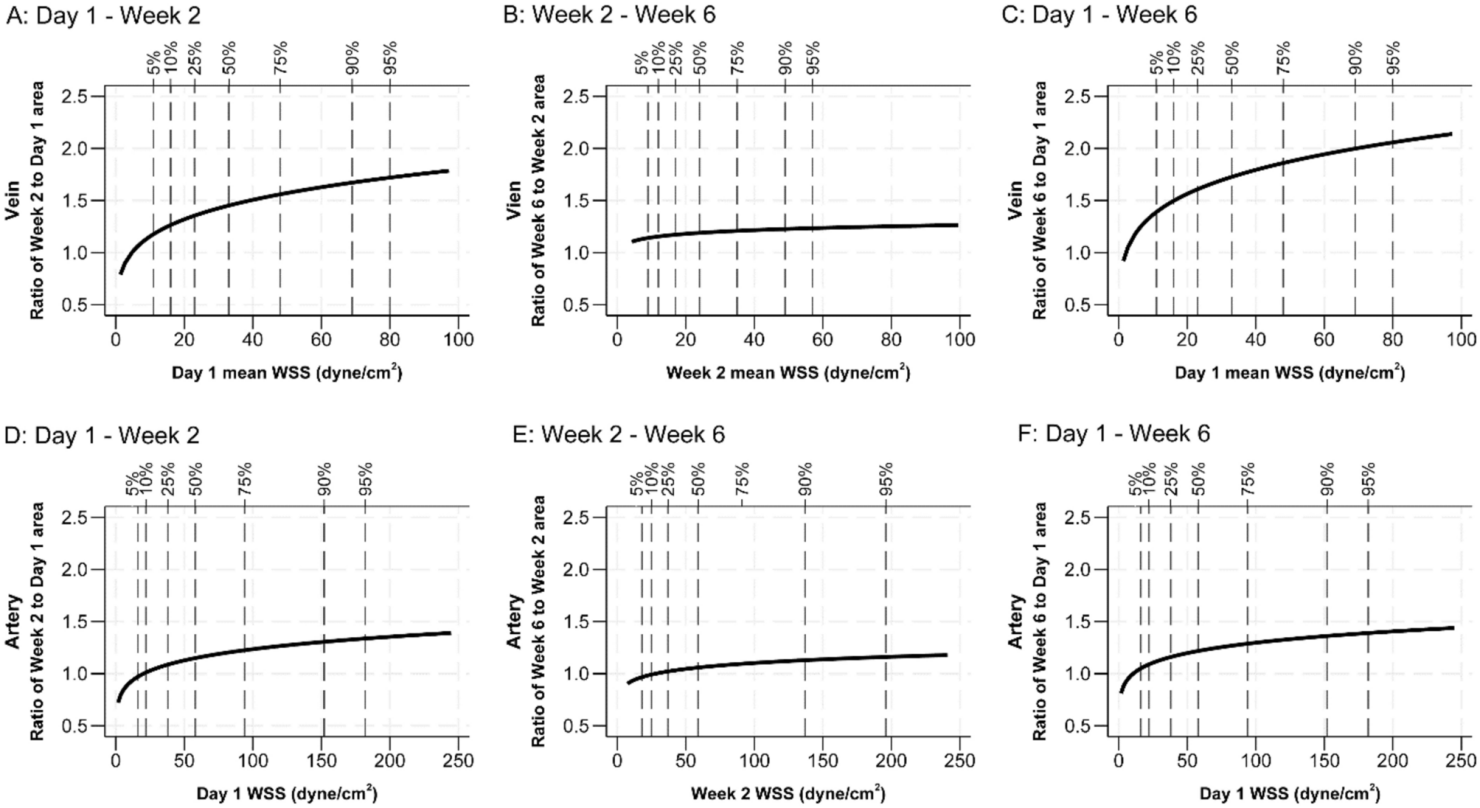
Associations between wall shear stress (WSS) and subsequent fistula lumen remodeling. Data on the vein are presented in panels **A**-**C**, while data on the proximal artery are presented in panels **D**-**F**. Plotted are lumen cross-sectional area ratio of Week-2 area to Day-1 area (**A** and **D**), Week-6 area to Week-2 area (**B** and **E**), and Week-6 area to Day-1 area (**C** and **F**) on y axis and the corresponding WSS in each period on the x axis. The 5^th^, 10^th^, 25^th^, 50^th^, 75^th^, 90^th^, and 95^th^ percentiles of WSS are labeled in each panel. Note that the plots do not show the entire range of WSS. The prediction is the result of a full linear-regression model (model IV) after logarithmic transformations of WSS and cross-sectional area ratios, adjusting for vein or artery diameter at the beginning of each period, baseline age, sex, race, diabetes, dialysis history, clinical center, preoperative artery and vein diameters, AVF location (forearm versus upper arm), flow-mediated dilation, and nitroglycerin-mediated dilation. A positive association between WSS and subsequent relative lumen area change was found for each time period for both vessels.

Statistically significant positive associations between WSS and subsequent relative lumen area changes were found for each time period in each of regression Models I-IV (Fig. 3; Table 4). The full regressions (Model IV) demonstrate that, with each doubling of WSS at Day 1, the fistula vein had a 10% greater increase in lumen area from Day 1 to Week 2 (P < 0.001) and a 9% greater increase from Day 1 to Week 6 (P < 0.001), but only a borderline non-significant 3% greater increase from Week 2 to Week 6 (P = 0.050). With doubling of WSS at the beginning of each period, the artery had a 4% greater area increase from Day 1 to Week 2 (P = 0.027), a 6% greater area increase from Week 2 to Week 6 (P = 0.002), and a 5% greater area increase from Day 1 to Week 6 (P = 0.020).

**Table 4:** Association of wall shear stress with subsequent lumen area ratios.

For each doubling of wall shear stress	Time period	Vessel	No adjustment (Model I)		Adjusting other covariates (Model II)^$^		Adjusting early diameter only (Model III)		Adjusting covariates and early diameter (Model IV)	
			Area ratio (95% CI)	P value	Area ratio (95% CI)	P value	Area ratio (95% CI)	P value	Area ratio (95% CI)	P value
At Day 1	Day 1-Week 2	Vein	1.16 (1.13, 1.20)	< 0.001	1.16 (1.13, 1.20)	< 0.001	1.11 (1.08, 1.14)	< 0.001	1.10 (1.07, 1.14)	< 0.001
		Artery	1.10 (1.08, 1.14)	< 0.001	1.10 (1.06, 1.15)	< 0.001	1.04 (1.00, 1.08)	0.035	1.04 (1.01, 1.09)	0.027
At Week 2	Week 2-Week 6	Vein	1.03 (1.00, 1.06)	0.024	1.03 (1.01, 1.06)	0.018	1.04 (1.01, 1.07)	0.016	1.03 (1.00, 1.06)	0.050
		Artery	1.08 (1.06, 1.11)	< 0.001	1.13 (1.09, 1.16)	< 0.001	1.07 (1.03, 1.11)	0.001	1.06 (1.01, 1.10)	0.002
At Day 1	Day 1-Week 6	Vein	1.16 (1.11, 1.20)	< 0.001	1.16 (1.11, 1.20)	< 0.001	1.11 (1.06, 1.15)	< 0.001	1.09 (1.05, 1.14)	< 0.001
		Artery	1.12 (1.09, 1.15)	< 0.001	1.12 (1.07, 1.17)	< 0.001	1.05 (1.00, 1.09)	0.032	1.05 (1.01, 1.10)	0.020

^$^The regression model adjusted for baseline age, sex, race, diabetes, dialysis history, clinical center, preoperative artery and vein diameters, and AVF location (forearm versus upper arm), flow-mediated dilation, and nitroglycerin-mediated dilation. The average area ratio was used for the vein.

We repeated these association analyses by replacing WSS in Table 4 with blood flow rate; the results of those analyses are presented in Table S5. As determined by the mathematical properties of regression modeling, the coefficients and P-values in Models III and IV adjusting for baseline diameter were identical to their counterparts for WSS in Table 4. However, without adjustment for baseline diameter, estimated lumen area ratios for vein flow rate were near 1.0 and not statistically significant in any time period in Models I and II and, for artery flow rate, were near 1.0 and only mildly statistically significant (P = 0.038 and 0.024, respectively) in Day 1-Week 2 or Day 1-Week 6 in Model I only. Moreover, the estimated ratios for both these periods were 0.96, reversing the direction of the arterial flow effect from that seen in either model including baseline diameter.

**Fig. 4:**
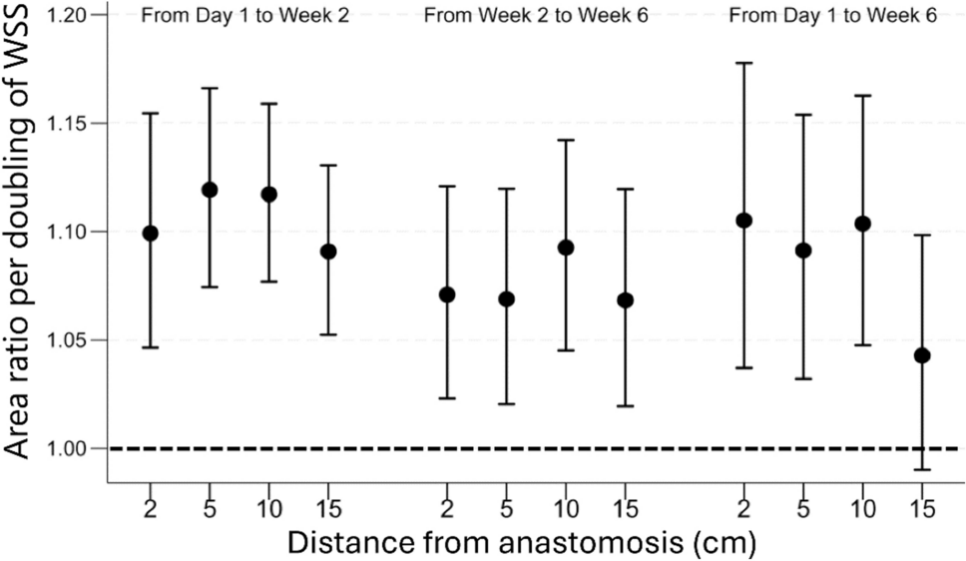
Vein lumen area changes at different locations from the anastomosis with each doubling of wall shear stress (WSS). The mixed effect models were adjusted for baseline age, sex, race, diabetes, dialysis history, clinical center, preoperative artery and vein diameters, arteriovenous fistula location (forearm versus upper arm), flow-mediated dilation, and nitroglycerin-mediated dilation. The area ratio and its 95% confidence interval are presented in the y axis.

We also compared the associations of WSS with subsequent vein area changes between the four vein measurement locations (Fig. 4). Without multiple comparison adjustment, the only statistically significant difference was between Day 1 to Week 6 changes at 10 and 15 cm from the anastomosis (P = 0.011), but this difference was not significant after Bonferroni multiple comparison adjustment.

### Associations of hemodynamics with clinical maturation

The number of AVFs with or without unassisted clinical maturation is 265 and 313, respectively. The AVFs without unassisted clinical maturation include those that achieved maturation after receiving intervention and those that never maturated. The diameter and flow rate of AVFs with unassisted clinical maturation at all three time points were larger than those without unassisted clinical maturation for both artery and vein respectively (Table S6). WSS quartiles at different time points according to the status of subsequent AVF unassisted clinical maturation are shown in Table 5. AVFs with successful unassisted clinical maturation had similar venous, but larger arterial, WSS than those without unassisted clinical maturation. The odds of unassisted maturation increased with increasing artery WSS in each of Models I-IV at each ultrasound examination, but with increasing vein WSS only in models adjusting for baseline diameter (Models III and IV, Table 6). In the fully adjusted Model IV, the odds of unassisted clinical maturation increased by 45% (95% CI: 11%-89%; P = 0.006) with each doubling of vein WSS at Day 1, and by 82% (95% CI: 39%-250%; P < 0.001) with each doubling of artery WSS at Day 1. For both vein and artery, in models in which the effect of WSS was statistically significant, the estimated odds ratio relating unassisted clinical maturation to WSS strengthened from Day 1 to Week 2 to Week 6.

**Table 5:** Vein or artery wall shear stress at various time points according to status of subsequent unassisted clinical maturation.

Time point	Vessel	Wall shear stress (dyne/cm^2^)		P value
		Mature	Not mature	
Day 1	Vein	32.3 (23.3, 45.1)	36.0 (22.7, 51.1)	0.73
	Artery	64.8 (43.4, 97.0)	50.9 (31.5, 81.5)	0.004
Week 2	Vein	23.9 (17.0, 33.5)	24.5 (16.6, 36.6)	0.66
	Artery	60.8 (44.2, 100.4)	53.7 (31.5, 87.2)	0.001
Week 6	Vein	21.5 (14.6, 31.7)	21.0 (13.1, 30.2)	0.17
	Artery	60.1 (42.4, 89.2)	47.4 (31.3, 74.9)	< 0.001

Presented are median wall shear stress values (25^th^ percentile, 75^th^ percentile) without imputation of missing values. P values were obtained from two-side t-test for log-scale of wall shear stress.

**Table 6:** Association of wall shear stress at various time points with subsequent unassisted clinical maturation.

For each doubling of wall shear stress	Vessel	No adjustment (Model I)		Adjusting other covariates (Model II)^$^		Adjusting early diameter only (Model III)		Adjusting covariates and early diameter (Model IV)	
		Odds ratio (95% CI)	P value	Odds ratio (95% CI)	P value	Odds ratio (95% CI)	P value	Odds ratio (95% CI)	P value
At Day 1	Vein	0.99 (0.81, 1.21)	0.94	1.03 (0.83, 1.29)	0.77	1.29 (1.02, 1.62)	0.032	1.45 (1.11, 1.89)	0.006
	Artery	1.19 (1.02, 1.40)	0.026	1.51 (1.20, 1.90)	< 0.001	1.71 (1.34, 2.17)	< 0.001	1.82 (1.39, 2.37	< 0.001
At Week 2	Vein	0.94 (0.76, 1.16)	0.58	0.97 (0.76, 1.23)	0.79	1.34 (1.04, 1.73)	0.023	1.49 (1.12, 1.99)	0.007
	Artery	1.23 (1.04, 1.46)	0.017	1.54 (1.22, 1.94)	< 0.001	2.26 (1.69, 3.03)	< 0.001	2.46 (1.79, 3.37)	< 0.001
At Day 1	Vein	1.19 (0.96, 1.47)	0.11	1.21 (0.96, 1.54)	0.11	2.11 (1.56, 2.84)	< 0.001	2.35 (1.67, 3.30)	< 0.001
	Artery	1.43 (1.18, 1.73)	< 0.001	1.94 (1.44, 2.61)	< 0.001	3.14 (2.12, 4.66)	< 0.001	3.22 (2.10, 4.92)	< 0.001

^$^The regression model adjusted for baseline age, sex, race, diabetes, dialysis history, clinical center, preoperative artery and vein diameters, and AVF location (forearm versus upper arm), flow-mediated dilation, and nitroglycerin-mediated dilation. The average area ratio was used for the vein.

We repeated these association analyses by replacing WSS in Table 6 with blood flow rate; the results of these analyses are presented in Table S7. Flow rate in both vein and artery at each time point was significantly associated with subsequent unassisted clinical maturation in all models, as expected since adequate flow rate is required for clinical maturation. As was necessarily so with the models for lumen area expansion, the odds ratios relating flow rate and WSS to unassisted maturation were identical when adjusting for the respective vessel diameter in each case (Tables 6 and S7).

## Discussion

In this prospective cohort study using parameters derived from routine duplex ultrasonography at prespecified vascular locations, we found positive associations of WSS with subsequent lumen expansion in both AVF vein and proximal artery in the first 6 weeks following AVF creation. This had been demonstrated in our prior study using more complex MRI and computational fluid dynamics simulation methods but with a much smaller group^22^. Taken together, these two studies are by far the most comprehensive assessment of the relationship between WSS and AVF remodeling. Additionally, in the present study we report a novel observation of positive associations between WSS and AVF clinical maturation, beyond lumen expansion.

By protocol design, the time points of AVF assessments differed between this ultrasound-based study (Day 1, Week 2, Week 6) and previous MRI-based study (Day 1, Week 6, Month 6). In combination, the two studies included AVF remodeling data at postoperative Day 1, Week 2, Week 6, and Month 6. The remodeling in the early Day 1-Week 2 period was the most dramatic, especially for the vein. The association between WSS and venous remodeling in this early period was also the strongest. From Week 2 to Week 6, vein lumen diameters increased slowly and the association between Week-2 WSS and Week-6 venous lumen expansion in the current study was marginally non-significant statistically. However, from Week 6 to Month 6, the association between WSS and venous remodeling in the previous study was statistically significant^22^. Arterial remodeling proceeded more gradually and the association between Week-2 WSS and arterial lumen expansion in Week 6 was statistically significant in the current study. Beyond Month 6, significant associations between WSS and lumen expansion are likely no longer present, as for vein bypass grafts for lower extremity occlusive arterial disease^20^.

**Fig. 5:**
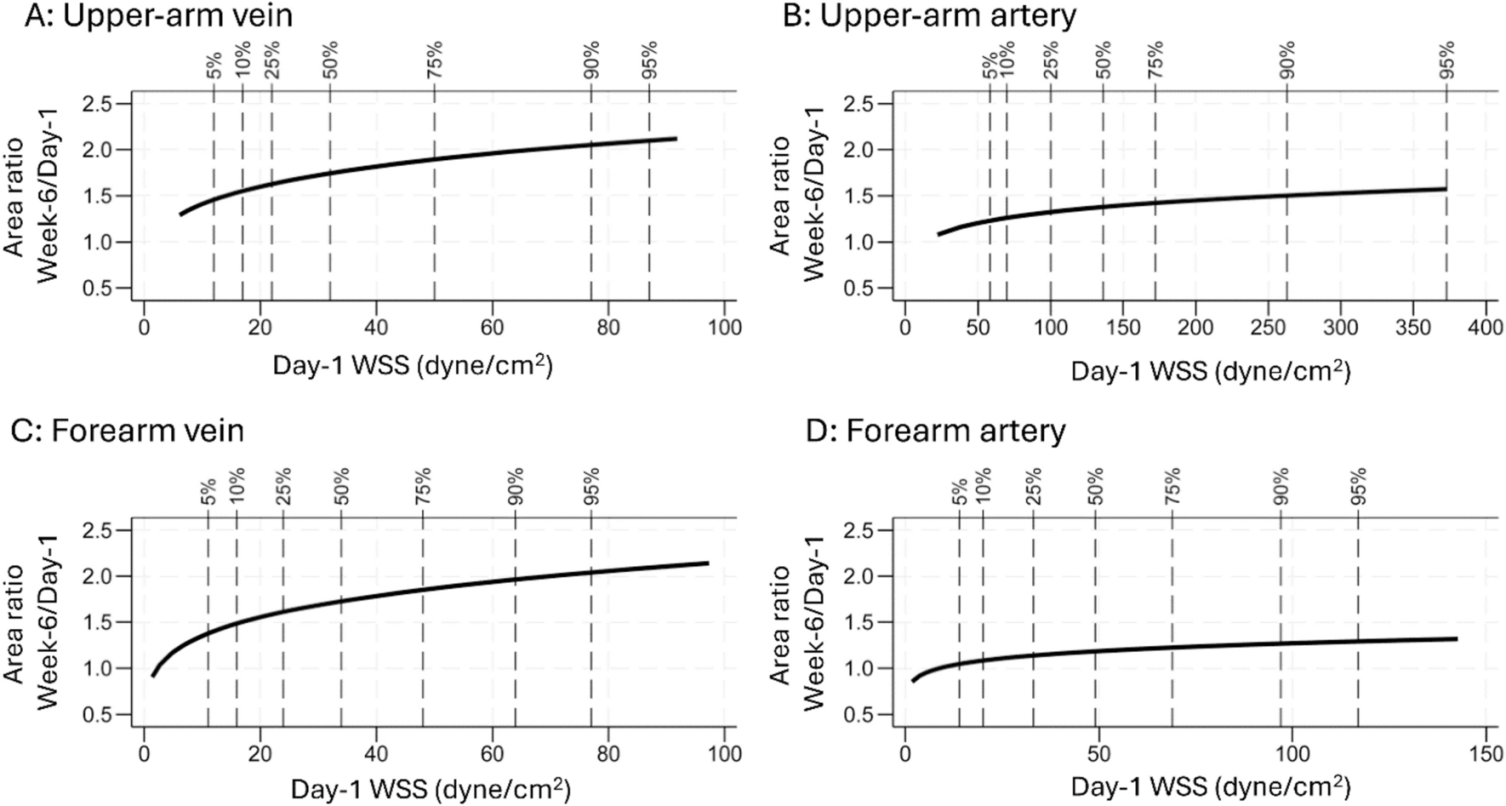
Associations between wall shear stress (WSS) and subsequent arteriovenous fistula lumen remodeling according to fistula location. Plotted are the predicted remodeling presented as lumen cross-sectional area ratio of Week-6 area to Day-1 area on y axis and corresponding WSS at Day 1 in the Day 1 to Week 6 period on x axis for vein (**A** and **C**) and artery (**B** and **D**). The 5^th^, 10^th^, 25^th^, 50^th^, 75^th^, 90^th^, and 95^th^ percentiles of WSS are labeled in each panel. The prediction is the result of a linear regression model after logarithmic transformations of WSS and cross-sectional area ratios, adjusting for vein or artery diameter at Day 1, baseline age, sex, race, diabetes, dialysis history, clinical center, preoperative artery and vein diameters, arteriovenous fistula location (forearm versus upper arm), flow-mediated dilation, and nitroglycerin-mediated dilation. Associations between WSS and lumen remodeling were not different between forearm and upper-arm AVFs for both vein and artery.

Upper-arm AVFs have a different overall maturate rate than forearm AVFs^28–30^. Therefore, associations between WSS and lumen expansion may be different between forearm and upper-arm AVFs. However, this concept cannot be confirmed in our previous MRI-based study^22^ or our current ultrasound-based study when the vein or artery diameter at the beginning of the studied duration was adjusted (Fig. 5). Forearm and upper-arm AVFs had similar positive associations between WSS and lumen expansion. Forearm and upper-arm AVFs had similar vein WSS (Table S3) although vein blood flow and diameter were different^8^. The HFM study also demonstrates that forearm and upper-arm fistulas have a similar rate of unassisted maturation^4^, which represents a more direct effect of WSS on maturation than overall maturation. The similar association between WSS and lumen expansion in our previous MRI-based study and current ultrasound-based study demonstrates the rigor of evidence supporting the association.

A positive association between WSS and subsequent lumen expansion supports the notion of endothelium-mediated outward remodeling under elevated flow^31^. Flow-induced endothelial responses range from instantaneous ion fluxes and biochemical signaling pathway activations to gene and protein expression. Shear stress-induced deformation at the endothelial cell surface is transmitted to multiple subcellular sites, including the nucleus, that are mechanically coupled to the cytoskeleton^32,33^. However, the knowledge of endothelial mechanotransduction is largely obtained from arterial studies in the setting of localized atherosclerosis. The cellular and molecular mechanisms of responses of AVF vein to altered hemodynamics are not well known, partly due to the lack of human AVF vein samples. One critical question regarding the newly created AVF is how much endothelial cells cover the luminal surface soon after AVF creation surgery. A recent study of a harvested AVF that required ligation due to steal syndrome found that the AVF vein had near complete coverage of endothelial cells 7 days after AVF creation^2^. Second-stage surgery of a two-stage upper-arm fistula provides a unique opportunity to obtain AVF tissue samples. A longitudinal transcriptomics study using bulk RNA sequencing to compare gene expressions of pre-access veins and the vein samples obtained at the second stage surgery reveals very important mechanisms of AVF responses to altered hemodynamics in patients^34^. Endothelial nitric oxide synthase (eNOS) is a central mediator of endothelial response to WSS. Interestingly, eNOS gene expression was statistically significantly higher in mature than nonmature AVF veins, and eNOS gene expression level was positively associated with larger AVF vein diameter. However, since WSS was not reported in that study, the associations between WSS and eNOS gene expression level and AVF remodeling are not known^34^. Combining the findings of this gene expression study with ours, we postulate that higher WSS increases eNOS activity and lumen expansion of AVF vein. Single-cell sequencing and spatial transcriptomics/proteomics techniques may be useful in further delineating the role of eNOS and other mediators in the AVF maturation process.

The positive association between WSS and subsequent lumen expansion suggests a positive association between WSS and subsequent fistula clinical maturation as well, which was indeed demonstrated in our analyses for both AVF artery and vein. A prior longitudinal, weekly study of a single patient over 15 weeks provided further evidence that WSS at the time of creation could be a useful predictor of AVF clinical maturation^35^. A larger WSS value would imply a larger flow rate at a similar lumen diameter, which is beneficial for successful fistula maturation. Considering the proven importance of WSS in arterial biology and pathophysiology, WSS provides a single mechanistic linkage of flow rate and lumen diameter to fistula remodeling and maturation.

Vein WSS was only statistically significantly associated with subsequent unassisted clinical maturation after controlling for lumen diameter at the time of the WSS value. However, artery WSS was significantly associated with maturation even without controlling for artery diameter. The reasons for this discrepancy are not known. One possible explanation is that the diameter of AVF vein needs to be big enough for successfully cannulation in order to achieve clinical maturation. Therefore, clinical maturation depends on the eventual absolute vein lumen diameter, which in turn depends on both the WSS-related lumen expansion ratio and the initial diameter as well. Combining with a previous finding that preoperative artery diameter, not vein diameter, is the more significant predictor of maturation^36^, the arterial quantities may be more useful for predicting unassisted clinical maturation.

Using the largest prospectively collected dataset so far, we explored the associations of postoperative WSS values with demographics and preoperative ultrasound measurements (Tables S3 and S4). Older age, female sex, black race, diabetes, and smaller preoperative diameter have been associated with worse fistula maturation and patency^4,5,30,37–39^. In our dataset, 10-year increase in age was associated with a slightly smaller WSS in both vein and artery while female and male participants had similar WSS in both vein and artery. Black participants had statistically significantly greater veinous, but similar arterial, WSS. Diabetic participants had similar veinous WSS but greater arterial WSS than non-diabetic participants. A larger preoperative vein diameter was not associated with a greater postoperative veinous, but a smaller arterial, WSS. Conversely, a larger preoperative artery diameter was associated with a smaller postoperative veinous, but a greater arterial, WSS. The largest pressure drop occurs at the anastomosis. The anastomosis cross-sectional area and angle affect this pressure drop, and subsequently the blood flow rate in an AVF^40^. A larger preoperative artery diameter would allow a surgeon to create an anastomosis with a larger cross-sectional area to reduce the pressure drop and increase flow rate. These relationships imply complex interactions between these factors and AVF remodeling and maturation.

There are, however, interpretational caveats with the use of WSS in the present study. As noted above, since WSS calculated from ultrasound data is proportional to the ratio of flow rate to a power of lumen diameter, in statistical models for any outcome that adjust for baseline lumen diameter, the regression coefficients for log(WSS) are numerically identical to the coefficients of log(flow rate) in analogous models where log(flow rate) replaces log(WSS). Also, in models without such adjustment that relate log(WSS) to subsequent change in lumen diameter, the coefficient of log(WSS) may be exaggerated because positive measurement error in lumen diameter at a baseline assessment reduces both apparent baseline log(WSS) and subsequent apparent change in lumen diameter. This creates spurious correlation from measurement error, a phenomenon sometimes known as “mathematical coupling”^41^. However, our MRI-based study, which did not have mathematical coupling, also demonstrated positive association between WSS and lumen remodeling^22^.

The large sample size and comprehensive prospectively collected data allow us to protect against potential confounding of the associations of WSS with AVF remodeling and maturation more adequately than previous and smaller studies. Limitations of this study include that determination of blood flow rates by duplex ultrasound and the formula for calculating WSS assume a parabolic flow profile for laminar flow, which is unrealistic for AVFs. To alleviate this effect, flow rate of the AVF vein was measured at 10 cm from the anastomosis, far away from the site with most flow disturbance. Flow rates of the AVF vein and proximal artery measured by ultrasound are generally comparable to those measured by phase-contrast MRI, which does not assume any flow profile^42^. Moreover, the fractions of missing preoperative flow-mediated dilation and, especially nitroglycerin-mediated dilation tests were relatively high because the nitroglycerin component was omitted if any of the baseline systolic blood pressures was less than 100 mmHg or the participant had a history of nitrate intolerance or migraine headaches or the participant had used sildenafil (Viagra), vardenafil (Levitra) or tadalafil (Cialis) in the past 7 days. Further analyses omitting the flow-mediated dilation and nitroglycerin-mediated dilation test results in the regression models demonstrated that the associations between hemodynamics and AVF remodeling and maturation were not significantly altered. Also, the use of flow rate and lumen diameter to estimate WSS makes the effect of WSS generally indistinguishable from that of flow rate in statistical models adjusting for diameter and, in models for lumen expansion without such adjustment, vulnerable to distortion by mathematical coupling. However, with ease of use and being in routine clinical practice, ultrasound can be used to more easily achieve a larger sample size in a longitudinal clinical study, just as the current study. Coupled with a prudent choice of covariates, ultrasound is a valuable research tool studying the associations between hemodynamics and vascular remodeling. Furthermore, as discussed previously, WSS, a specific combination of flow rate and lumen diameter, is believed to biologically mediate vascular responses to altered flow rate^31^, and thus contributes to AVF lumen remodeling. Finally, although high WSS has been reported to promote atherosclerotic plaque rupture and thrombosis^43^, once the fistula is used for routine dialysis, frequent needle cannulations cause vascular injury unrelated to WSS. Moreover, the HFM study investigated factors associated with AVF maturation and did not collect late AVF failure data. Hence, the relationship of WSS to late AVF failure was not investigated in this study.

## Conclusion

WSS was positively associated with subsequent expansion of the AVF vein and proximal artery during early remodeling of AVF and unassisted clinical maturation, even after statistical adjustments for multiple covariates. Strategies that promote endothelial response to increased WSS may improve AVF maturation outcomes.

## Supplementary Information

Supplementary Information.

## Data Availability

The data can be requested via NIDDK Central Repository via https://repository.niddk.nih.gov/studies/hfmc.
