# Supplementary material for: Hemodynamics are associated with subsequent lumen remodeling and clinical maturation of hemodialysis arteriovenous fistula
Source: Sci Rep. 2025 Feb 19;15:6131. doi: 10.1038/s41598-025-89896-z (PMC11840087; doi:10.1038/s41598-025-89896-z)
Supplement: Supplementary file 1 — Supplementary Information. [file 41598_2025_89896_MOESM1_ESM.pdf]

Supplementary Material

Table S1. Preoperative vascular mapping and function test with imputation data

	With imputation	Raw
PRE-OP minimal vein diam (mm)	3.0 (1.2)	3.0 (1.1)
PRE-OP average vein diam (mm)	3.7 (1.2)	3.7 (1.2)
PRE-OP artery diam (mm)	3.8 (1.2)	3.8 (1.2)
Brachial artery FMD (%)	4.7 (4.9)	4.8 (5.0)
Brachial artery NMD (%)	7.5 (7.4)	7.2 (6.3)

Data are mean (SD). The raw data have been presented in the main text and are included here for the comparison purpose. Number of imputations: 10. Number of missing data: PRE-OP minimal vein diam = 12; PRE-OP average vein diam = 12; PRE-OP artery diam = 9; FMD = 53; NMD = 142.
PRE-OP: preoperative; diam: diameter: FMD: Flow-mediated dilation; NMD: nitroglycerin-mediated dilation

Table S2 Postoperative fistula flow rate data with imputation information

Flow rate (mL/min)	With imputation	Raw
Fistula vein at Day 1	686 (429)	692 (434)
Fistula vein at Week 2	897 (515)	894 (519)
Fistula vein at Week 6	1008 (617)	1026 (621)
Fistula proximal artery at Day 1	788 (432)	794 (433)
Fistula proximal artery at Week 2	984 (490)	981 (491)
Fistula proximal artery at Week 6	1072 (563)	1068 (552)

Data are mean (SD). The raw data have been presented in the main text and are included here for the comparison purpose. Number of missing flow rate data: Fistula vein at Day 1 = 15; at Week 2 = 32; at Week 6 = 46.
Fistula proximal artery at Day 1 = 15; at Week 2 = 32; at Week 6 = 46.

Table S3. Percent change of the mean wall shear stress values of the vein with demographics and preoperative ultrasound measurements

	Change (%)	P value	Change (%)	P value	Change (%)	P value
Time	Day1		Week 2		Week 6	
Per 10 year increase in age	-3.8 (-7.9, 0.4)	0.08	-4.2 (-7.8, -0.5)	0.027	-2.7 (-6.8, 1.6)	0.21
Female vs male	-2.6 (-14.3, 10.7)	0.68	-0.7 (-11.0, 10.8)	0.9	-8.4 (-19.2, 3.8)	0.17
Black race vs other races	17.4 (4.8, 31.5)	0.006	14.4 (3.2, 26.9)	0.011	12.9 (0.8, 26.4)	0.036
Diabetes vs non-diabetes	3.2 (-7.7, 15.3)	0.58	5.6 (-4.6, 17.0)	0.29	-3.0 (-13.6, 9.0)	0.61
On dialysis vs not on dialyasis	-11.9 (-21.0 -1.7)	0.024	-9.1 (-17.7, 0.4)	0.06	-7.6 (-17.3, 3.2)	0.16
Forearm vs upper-arm fistula	9.1 (-10.3, 32.6)	0.38	-6.0 (-20.9, 11.7)	0.48	-7.2 (-23.7, 12.8)	0.45
Per 1 mm increase in preop vein diameter	0.8 (-6.2, 8.3)	0.83	5.0 (-1.7, 12.2)	0.15	-0.4 (-7.2, 6.9)	0.92
Per 1mm increase in preop artery diameter	-12.0 (-16.3, -7.6)	<0.001	-10.1 (-14.0, -6.1)	<0.001	-8.2 (-12.2, -3.9)	<0.001
Per 1% increase in NMD	2.2 (-9.1, 15.0)	0.71	1.7 (-8.2, 12.7)	0.75	-3.4 (-14.6, 9.1)	0.57
Per 1% increase in FMD	-2.6 (-14.8, 11.3)	0.70	-2.6 (-13.7, 9.8)	0.66	2.8 (-9.9, 17.2)	0.68
Clinical center number (center 1 as reference)						
2	29.8 (6.4, 58.2)	0.01	18.1 (-1.7, 41.9)	0.08	7.6 (-12.0, 31.6)	0.47
3	27.3 (8.8, 49.0)	0.003	27.7 (11.1, 46.8)	<0.001	16.9 (0.1, 36.6)	0.05
4	28.6 (6.8, 54.7)	0.008	30.2 (11.4, 52.3)	0.001	21.8 (2.3, 45.0)	0.027
5	23.4 (3.3, 47.3)	0.021	9.7 (-6.9, 29.3)	0.27	-5.9 (-21.4, 12.7)	0.51
6	5.5 (-18.1, 36.0)	0.68	4.2 (-17.4, 31.4)	0.73	-19.0 (-36.4, 3.2)	0.09
7	8.9 (-13.8, 37.5)	0.48	31.0 (5.8, 62.1)	0.014	-2.8 (-24.0, 24.3)	0.82

The change (%) data are mean (95% confidence interval). NMD: nitroglycerin-mediated dilation; FMD: Flow-mediated dilation

Table S4. Percent change of the wall shear stress values of the artery with demographics and preoperative ultrasound measurements

	Change (%)	P value	Change (%)	P value	Change (%)	P value
Time	Day1		Week 2		Week 6	
Per 10 year increase in age	-7.4 (-11.3, -3.3)	<0.001	-2.6 (-6.7, 1.8)	0.24	-6.8 (-10.4, -2.8)	0.001
Female vs male	2.9 (-9.6, 17.0)	0.67	-4.2 (-15.5, 8.6)	0.5	-4.2 (-16.1, 9.2)	0.52
Black race vs other races	-9.2 (-19.5, 2.4)	0.12	-3.1 (-13.9, 8.9)	0.6	-3.3 (-13.5, 8.2)	0.56
Diabetes vs non-diabetes	18.5 (4.6, 34.2)	0.008	30.4 (16.7, 45.7)	<0.001	28.5 (15.1, 43.5)	<0.001
On dialysis vs not on dialyasis	0.6 (-10.3, 12.9)	0.91	-2.8 (-13.1, 8.8)	0.62	-0.3 (-10.5, 11.0)	0.96
Forearm vs upper-arm fistula	-53.5 (-61.6, -43.6)	<0.001	-43.1 (-53.6, -30.2)	<0.001	-40.9 (-51.2, -28.4)	<0.001
Per 1 mm increase in preop vein diameter	-17.6 (-23.0, -11.7)	<0.001	-19.3 (-24.8, -13.3)	<0.001	-20.1 (-25.6, -14.2)	<0.001
Per 1mm increase in preop artery diameter	8.0 (3.0, 13.3)	0.002	7.6 (2.6, 12.8)	0.003	10.5 (5.6, 15.6)	<0.001
Per 1% increase in NMD	-7.5 (-19.7, 6.4)	0.27	-2.0 (-12.7, 10.0)	0.73	-9.6 (-18.5, 0.3)	0.06
Per 1% increase in FMD	-2.9 (-17.1, 13.7)	0.71	-6.7 (-18.7, 7.2)	0.33	-4.6 (-15.7, 8.0)	0.46
Clinical center number (center 1 as reference)						
2	70.7 (39.5, 108.8)	<0.001	43.4 (16.2, 77.0)	<0.001	19.9 (-2.1, 46.7)	0.08
3	56.1 (32.6, 83.7)	<0.001	40.6 (19.5, 65.5)	<0.001	16.4 (-0.1, 35.7)	0.05
4	41.9 (18.5, 70.1)	<0.001	27.9 (7.3, 52.4)	0.006	8.3 (-9.2, 29.1)	0.38
5	34.0 (11.8, 60.8)	0.002	11.3 (-7.4, 33.8)	0.26	6.3 (-11.0, 26.9)	0.5
6	27.7 (-1.2, 65.1)	0.06	28.1 (0.7, 63.1)	0.045	10.2 (-12.7, 39.3)	0.41
7	-10.9 (-32.3, 17.3)	0.41	-15.2 (-33.0, 7.5)	0.17	-27.2 (-42.8, -7.3)	0.01

The change (%) data are mean (95% confidence interval). NMD: nitroglycerin-mediated dilation; FMD: Flow-mediated dilation

Table S5. Association of flow rate with subsequent lumen area ratio when adjusting different variables

For each doubling of flow rate	Time period	Vessel	No adjustment (Model I)		Adjusting other covariates (Model II)[$]		Adjusting early diameter only (Model III)		Adjusting covariates and early diameter (Model IV)	
			Area ratio (95% CI)	P value	Area ratio (95% CI)	P value	Area ratio (95% CI)	P value	Area ratio (95% CI)	P value
At Day 1	Day 1-Week 2	Vein	0.98 (0.96, 1.01)	0.17	1.01 (0.97, 1.04)	0.74	1.11 (1.08, 1.14)	<0.001	1.10 (1.07, 1.14)	<0.001
		Artery	0.96 (0.93, 1.00)	0.038	0.99 (0.95, 1.03)	0.50	1.04 (1.00, 1.08)	0.035	1.04 (1.01, 1.09)	0.027
At Week 2	Week 2-Week 6	Vein	1.02 (1.00, 1.04)	0.09	1.01 (0.99, 1.04)	0.37	1.04 (1.01, 1.07)	0.016	1.03 (1.00, 1.06)	0.050
		Artery	1.00 (0.97, 1.04)	0.85	1.01 (0.97, 1.04)	0.79	1.07 (1.03, 1.11)	0.001	1.06 (1.01, 1.10)	0.002
At Day 1	Day 1-Week 6	Vein	0.98 (0.95, 1.01)	0.24	0.99 (0.95, 1.03)	0.74	1.11 (1.06, 1.15)	<0.001	1.09 (1.05, 1.14)	<0.001
		Artery	0.96 (0.92, 0.99)	0.024	0.98 (0.94, 1.03)	0.43	1.05 (1.00, 1.09)	0.032	1.05 (1.01, 1.10)	0.020

[$]The regression model adjusted for baseline age, sex, race, diabetes, dialysis history, clinical center, preoperative artery and vein diameters, and AVF location (forearm versus upper arm), flow-mediated dilation, and nitroglycerin-mediated dilation. The average area ratio was used for the vein.

Table S6. Diameter and flow rate of fistulas with or without unassisted maturation

Time	Vessel	Diameter (mm)		Flow rate (mL/min)	
		Mature	Not mature	Mature	Not mature
Day 1	Vein	5.1 (1.3)	4.6 (1.1)	792 (447)	607 (392)
	Artery	4.3 (1.1)	4.2 (1.2)	921 (463)	718 (380)
Week 2	Vein	6.2 (1.4)	5.4 (1.2)	1035 (541)	757 (459)
	Artery	4.6 (1.1)	4.5 (1.1)	1137 (502)	874 (464)
Week 6	Vein	6.9 (1.7)	5.9 (1.4)	1245 (638)	797 (525)
	Artery	4.8 (1.1)	4.6 (1.2)	1261 (546)	924 (537)

Data are mean (SD).

Table S7. Association of flow rate with unassisted clinical maturation when adjusting different variables

For each doubling of flow rate	Vessel	No adjustment (Model I)		Adjusting other covariates (Model II)$		Adjusting early diameter only (Model III)		Adjusting covariates and early diameter (Model IV)	
		Odds ratio (95% CI)	P value	Odds ratio (95% CI)	P value	Odds ratio (95% CI)	P value	Odds ratio (95% CI)	P value
At Day 1	Vein	1.55 (1.29, 1.85)	<0.001	1.79 (1.42, 2.26)	<0.001	1.29 (1.02, 1.62)	0.032	1.45 (1.11, 1.89)	0.006
	Artery	1.62 (1.33, 1.97)	<0.001	1.78 (1.39, 2.27)	<0.001	1.71 (1.34, 2.17)	<0.001	1.82 (1.39, 2.37	<0.001
At Week 2	Vein	1.80 (1.47, 2.21)	<0.001	2.16 (1.69, 2.77)	<0.001	1.34 (1.04, 1.73)	0.023	1.49 (1.12, 1.99)	0.007
	Artery	2.09 (1.63, 2.67)	<0.001	2.39 (1.78, 3.22)	<0.001	2.26 (1.69, 3.03)	<0.001	2.46 (1.79, 3.37)	<0.001
At Day 1	Vein	2.39 (1.90, 3.02)	<0.001	2.99 (2.22, 4.03)	<0.001	2.11 (1.56, 2.84)	<0.001	2.35 (1.67, 3.30)	<0.001
	Artery	2.50 (1.87, 3.34)	<0.001	2.81 (1.98, 4.00)	<0.001	3.14 (2.12, 4.66)	<0.001	3.22 (2.10, 4.92)	<0.001

$The regression model adjusted for baseline age, sex, race, diabetes, dialysis history, clinical center, preoperative artery and vein diameters, and AVF location (forearm versus upper arm), flow-mediated dilation, and nitroglycerin-mediated dilation. The average area ratio was used for the vein.
